# Prosocial behaviour axioms and values: Influence of gender and volunteering

**DOI:** 10.1186/s41155-023-00258-y

**Published:** 2023-07-10

**Authors:** María del Carmen Olmos-Gómez, Francisca Ruiz-Garzón, David Azancot-Chocron, Rafael López-Cordero

**Affiliations:** 1 Department of Methods of Research and Diagnosis in Education, University of Granada, Granada, Spain; 2 Department of Didactics of Social Science, University of Granada, Granada, Spain

**Keywords:** Values, Prosocial Behaviors, Gender, Social Volunteering, Multicultural Context

## Abstract

To promote prosocial behaviour, in the present study, we observed the human values that may predict it within the realms of the transcendental views of life, society’s shared culture and the world of personal and interpersonal affections. To do this, we started with two hypotheses: (1) prosocial behaviour differs according to gender and participation in volunteering; and (2) the variables of transcendental values, cultural development, affective development, gender and participation in volunteering predict prosocial behaviour.

To do so, we conducted a quantitative study based on the cross-sectional, social analytical-empirical research method. We used a validated instrument with a large sample of 1,712 individuals living in the multicultural context of the Spanish city of Melilla, located in North Africa and one of only two land borders between Europe and Morocco. Values that could promote prosocial behaviour were grouped into four dimensions to locate relevant factors that helped identify which values are linked to specific actions, both formal and informal, through an inferential analysis focusing on regression and multivariate analysis of variance.

Our findings highlighted the linkage of the transcendent dimension of the individual in relation to his or her level of prosocial behaviour and the role of women as socialising agents.

## Introduction

Prosocial behaviours are those performed voluntarily to benefit others. These behaviours are understood as desirable, beneficial and effective for society. The measurement of prosocial behaviour requires an orderly and updated knowledge base, which helps to identify it (Marti-Vilar et al., 2019). There is uncertainty as to what is to be regarded as intuitive: prosocial or antisocial behaviour? To answer this question, Gallotti and Grujic (2019) found that although initially the intuitive decision of people is to cooperate, rational deliberation quickly prevails over an initial intuitive bias towards cooperation. This is fostered by positive interactions, and it is frustrated by negative ones. However, this initial prosocial tendency is resilient because after a pause, it resets to the same initial value.

From an early age, children are motivated to help others. In their study of the internal biological mechanisms underlying young children’s motivation to help others, Hepach et al. (2019) suggested that their propensity to help is directly related to their physiological arousal after witnessing that others need help. However, studies such as those by Lichtenberg et al. (2018) and their experiments to understand the neural foundations of the link between behaviour and dopamine release reflect a subjective rather than an objective assessment.

In the search for prosocial values, although there have been studies on the relationship of parental reinforcement and parental values to prosocial behaviour in young children (Eisenberg et al., 1992), there have not been many recent studies on the direct relationship between values and prosocial behaviour.

Ortiz Baron et al. (2018) found that prosocial behaviour was predicted by the interaction between empathy and moral pride, by guilt and, to a lesser extent and negatively, by shame. In relation to antisocial behaviour, children who are particularly prone to feeling guilty scored lower on antisocial behaviour, regardless of their level of empathy. Nevertheless, the combination of low empathy and low levels of guilt was associated with higher scores for antisocial behaviour. Shame was moderately associated with antisocial behaviour.

Balabanian and Lemos (2020), in their study on attributional patterns among adolescents, confirmed the idea that mental models based on beliefs about the world have a decisive influence on the tendency to be prosocial. They found that those who chose internal, controllable factors as their causal explanation were less likely to be prosocial, since attributing responsibility for the situation to the subject who suffers from it would have an unfavourable effect on the performance of helping actions. The mental models about the reality that surrounds us are linked to the concept of transcendence.

Transcendence is related to what is beyond the natural world. The transcendent is associated with the immortal and the essential. To transcend is to excel, to reach in some way that which lies outside the limits imposed by the body. Barton and Hart (2023) present self-transcendence as a set of values and a state of mind that can enhance motivation to engage in social activism. This involves an effort to connect to a larger context with a prosocial intent to serve the greater good. Self-transcendence is defined as “increased awareness of dimensions greater than the self and the expansion of personal boundaries within intrapersonal, interpersonal, transpersonal and temporal domains”.

It is crucial to highlight the importance of the cultural and social context. When studying the relationship between the mechanisms of moral disconnection, empathy, and prosociality in delinquent adolescents, Gómez and Narváez (2019) concluded that it is important to encourage empathy, solidarity, emotional education, and prosocial moral criteria in juvenile offenders. Gómez-Tabares (2019), in analysing prosocial tendencies and their relationship with empathy and self-efficacy beliefs for the regulation of affection, found that emotional factors, especially in emergencies, obedience, or complacency, are related to prosocial expressions. When re-encounters are likely, developing a positive reputation can be an asset that will lead to better results. However, in real life, acts of cooperation are ambiguous and occur in noisy environments in which individuals may have multiple goals, visibility is reduced, and reputation systems may be different. Duradoni et al. (2018) suggested that reputational effects increase fairness and trust even in noisy, ambiguous, and uncertain environments. Collodi et al. (2020) claimed that reputation seems to decrease impartiality in individuals who report high levels of neuroticism and openness. Despite this, high self-efficacy values seem to be more likely linked to unfair behaviour when reputation is not part of the negotiation. Age and sense of community emerge as promoters of fairness regardless of the experimental condition. Guilt leads people to develop prosocial behaviours, but the effects of guilt can also be counterproductive. In this regard, Graton and Mailliez (2019) advocated that attentional biases are better predictors of the effectiveness of a message than the amount of emotion induced by the same message.

In turn, Christov-Moore et al. (2014) reviewed several studies and showed that there are fundamental differences in implicit measures of empathy. Empathic behaviour appeared to be particularly strong in social species with prolonged parental care, such as mammals and some birds. It has phylogenetic and ontogenetic roots in biology, so it is not a mere cultural byproduct of socialisation. Work with animals has shown that individuals prefer to behave prosocially and cooperatively, with sex differences reported for a diverse number of behaviours, making a convincing case that females possess higher levels of empathy than males, at least in some species. If such sex differences were exclusively culturally determined, this would imply that animals also transmit cultural gender expectations.

Empathy and gender are difficult to define, as the disciplines that study them often use different terminologies and methods. Some authors suggest that part of the observed gender differences may be due to cultural expectations about gender roles. Some studies claim that women are more empathetic when they are aware that they are being evaluated and that neither gender nor moral judgements seem to be good predictors of empathy (Baez et al., 2017). Although previous studies have proven that the moral principle of caring for others is associated with giving to charitable organisations and mediates the empathic concern-giving relationship, the principle of care seems to play a more important role than empathy (Bekkers & Ottoni-Wilhelm, 2016).

Women also tend to exhibit more prosocial and altruistic behaviour, which supports the idea that affective empathy drives prosocial behaviour. Christov-Moore et al. (2014) argue that there are social, contextual and cultural factors that may influence some of these observed behavioural and neural differences in affective empathy between men and women. Particularly in adulthood, it seems that men vary more than women in certain aspects of emotional processing and altruistic behaviour. Although men appear to express less empathy, their higher discrimination in targeting helping behaviour supports the idea that men actually outperform women in their empathic control. In fact, even in childhood, men seem to have more control over their empathy than women. The results of the study by Paulin et al. (2014) indicate that women tend to be more altruistic, empathic and moral than men, pointing to the importance of understanding the challenge of engaging men emotionally in prosocial behaviour.

Gatley (2021) argued that the intrinsic/extrinsic value distinction has little to do with the educational value of an activity. The distinction between instrumental and final value is relevant, but it does not imply that activities with final value are more valuable from an educational standpoint than those having an instrumental value. However, values do not always help to promote prosocial behaviour. Young and West (2010), when investigating the possible relationship between values and substance use in adolescence, concluded that the evidence from their study did not support the argument that the possession of certain “prosocial” or “good” values substantially protects against such use and questioned the efficacy of value-based interventions in relation to substance use.

Describing behaviours as a reflection of categories (for example, asking children to “be helpers”) has been shown to increase prosocial behaviour. In this regard, Foster-Hanson et al. (2020) showed that such effects are counterproductive if children experience setbacks while performing category-relevant actions and discussed the implications of how category labels shape beliefs and behaviour. The category of helper can be linked to the concept of volunteering. Volunteering is understood by Lopez-Cordero (2020) as a form of altruistic behaviour that aims to offer help to others, to a group, to an organisation, to a cause, or to the community in general, without the expectation of a subsequent material reward. In relation to volunteering, studies such as Wiepking, Einolf and Yang (2022) showed that gender has a direct effect on volunteering. In addition, gender differences in volunteering vary by country, which could indicate that these differences may have a social rather than a biological basis (Einolf, 2011).

Regarding prosocial principles, moral “nudges,” which are based on socially accepted values, can promote prosocial behaviour. These are especially important because they are inexpensive and easy to apply. These mechanisms can change people’s behaviour without prohibiting any options or significantly changing their financial incentives. Tyers (2018) examined the application of “nudges” to promote prosocial behaviour, concluding that they are unlikely to be effective when the target behaviour is not perceived as common, is not visible, and has negative connotations. Capraro et al. (2019) explored whether moral “nudges” promoted donations to humanitarian organisations and found that they increased donations by 44%. They also showed that asking individuals to declare “what they believe is morally correct” not only increased prosociality in their immediate future choices but also in subsequent choices, even when the social context changed.

Incentive-induced prosocial behaviours that promote “good” causes by influencing socially relevant decisions in desirable ways, for example, by increasing pro-environmental options or prosocial behaviour in general, are remarkably successful when judged by their effects on the targeted behaviours in isolation. Even in view of the fear that the indirect effects of the behaviour could eliminate or even reverse the initially positive effects of choice defaults, Ghesla et al. (2019) argued that the prosocial behaviour induced by choice defaults has no adverse effects on subsequent behaviour. In terms of social recognition, Futamura (2018) stresses the importance of performing prosocial actions on a regular basis and not only on an exceptional basis. Extraordinary prosocial acts are highly valued when they are accompanied by ordinary prosocial behaviour.

In view of the above, this research study has two main objectives. First, we analyse prosocial behaviour, as well as the values of transcendence, culture and affection, according to the variables of gender and participation in volunteering. Second, we aim to identify which dimensions of transcendence, culture and affection predict prosocial behaviour.

In addition, different hypotheses are proposed based on the findings of the research studies included in the previous literature review. Hypothesis 1: Prosocial behaviour differs according to gender and participation in volunteering. Hypothesis 2: The variables of transcendental values, cultural development, affective development, gender and participation in volunteering predict prosocial behaviour.

## Materials and methods

### Participants

For this research, a quantitative study was conducted based on the social analytical-empirical research method. Nonprobability sampling was used. The sampling was carried out with online access to the questionnaire through the public bodies that allowed us to send the questionnaire (city councils, educational centres, nongovernmental organisations, cultural centres, etc.). A sample of 1,712 individuals aged 16–45 years was examined in the study. The characteristics of the sample were determined by the place where the questionnaire was administered; a Spanish enclave in North Africa called the autonomous city of Melilla, where one of the only two land borders between Europe and Morocco is located. The population living there is characterised by a high number of civil servants, a large number of military personnel, and workers of small or medium-sized companies. Together with low fiscal pressure, this gives rise to a high economic capacity that coexists with a cross-border population of a very low economic level. This produces a patent social inequality (Echeverria, 2021), considering that the origin of the respondents and their religion are essential elements for the study of values in cross-border multicultural contexts to better understand their possible influence on prosocial behaviour. The description of the participants addressed their sociodemographic characteristics (gender, age, religion, whether they had children, whether they had dependents, and whether they were volunteers). The respondents with European cultural origins were mainly Spaniards and were Catholic. They were born in the autonomous city of Melilla or elsewhere in the Iberian Peninsula but had changed residence for professional or family reasons. Children with Amazigh cultural origins born in the autonomous city of Melilla, and therefore Spaniards, were mostly Muslims and had families in Morocco or other neighbouring areas. As Melilla borders Morocco, they often travelled to visit relatives in nearby Moroccan areas (Rif region in Morocco). There was also a remarkable number of Jews from Melilla, families who, although settled in Melilla, came from different areas. They were a substantial community in the city, especially in the business sector.

The mean age of the participants was 24.42 years, with a standard deviation of 7.481 and a distribution of 65.9% women and 34.1% men. Regarding marital status, 9.2% were married, 1.2% were divorced, 76.3% were single and 0.4% were widowed. A total of 12.9% of participants did not answer this question. The level of education of the sample was as follows: 2.8% had a primary education, 25.4% had a secondary education, 57.5% were graduates, 0.8% were postgraduates (PhD), and 13.5% did not answer. Concerning religion, 56.5% were Christian, 20.2%, Muslim, 0.8%, Jewish, 0.2%, Hindu, and 20.8% had no religion; 69.6% were volunteers, and 30.4% were not; 27.4% were caring for elder or disabled family members, and 70.9% were not; 78.1% had children, and 21.9% did not.

## Procedure

This study was developed in accordance with current privacy and data protection regulations. This implies that the participants gave their informed consent to those in charge of the study to process their personal data in accordance with the provisions of Regulation (EU) 2016/679, April 27th (GDPR), and the Organic Act no. 3/2018, December 5th (LOPDGDD). This research project was carried out according to the guidelines of the Declaration of Helsinki and later updated in Brazil in 2013 and was approved by the Ethics Committee of Psychoeducational Research of the University of Granada (201–300 Academic Ranking of World Universities, Shanghai, 2020). And it was carried out according to the International Test Commission guidelines. It was also approved by the academic commission of Social Responsibility of the Faculty of Education of the University of Granada. The participants were informed of the objectives, purpose, and benefits of the research and of the commitment to anonymity.

The questionnaire was conducted online through Google Forms.

### Instrument

To achieve the objectives and test the hypotheses proposed, we designed, developed and tested the psychometric properties of an ad hoc questionnaire, which was later used to obtain the data.

The design of the items was based on various instruments developed by Gervilla (2004), “Searching for values: Axiological content analysis” and the updated version by González et al. (2021) “Analysis and validation of a test to measure values”. On the basis of these two instruments, the items were developed, and the psychometric properties were evaluated.

To assess the adequacy, consistency and coherence of the questions, as well as their relevance, a rating scale showing a level of agreement from 1 to 3 for each of the questions was designed. A three-round Delphi study was conducted with experts in the field (Vidal & Lluch, 2019) to validate the content (Escobar & Cuervo, 2008). Apart from that, each question allowed the possibility of including modifications, suggestions or comments in an open response section. (Almonacid-Fierro et al., 2018). The panel of experts included seven university professors of psychology (4), education (2) and social sciences (1). After the completion of three rounds, the level of agreement on each question was analysed, and the final questionnaire was elaborated, removing and modifying those questions with a percentage of agreement below 2. Eventually, the final version of the questionnaire, which can be found in the Appendix of this paper, was completed with an agreement rate of K = 0.87.

The content validation phase was completed with the statistical calculations of the exploratory and confirmatory analysis, as well as with the calculation of the reliability of the final instrument that was used in this study. The aspects related to the psychometric analysis are explained below.

### Validation and reliability of the instrument

Content analysis was used to assess the items within the questionnaire. The internal consistency index, or Cronbach’s alpha, calculated for this study had a value of α = 0.879, and its factorial or construct validity was corroborated by other research (Elosua & Muñiz, 2010; Malo et al., 2016). In addition, quantitative data were analysed according to descriptive statistics and internal consistency estimates. For this purpose, SPSS 25.0 was used, as mentioned above. To determine these factors, a structure correlation analysis was employed, and an orthogonal rotation factor analysis was conducted, thereby determining which group of variables had a high correlation with each factor. The data were tested to determine the type of statistical procedure to be adopted (parametric or nonparametric tests). First, the normality of the data was examined by checking the skewness and kurtosis values of the different items that made up the questionnaire. In this case, no high dispersion was obtained, assuming normality in the data and opting for parametric tests. Second, parametric tests were used since the assumption of homogeneity of variance was met as Levene's test and the sample size was sufficiently large (Andréu, 2011).

Model confirmation was performed using structural equation modelling (SEM). Then, we grouped the items (observed variables) into 4 factors as unobserved exogenous variables. The identification of the groupings of factors in four dimensions included Sociability (SOC) (the tendency to deal with and relate to people), Transcendence (TRA) (addressing relationships with realities perceived as beyond natural limits), Culture (CUL) (consideration of the ways of life and customs, artistic development, and expressions of the social group to which one belongs) and Affection (AF) (referring to mood and emotion tendencies). The values obtained that verified the validity of the model were the following: normalised fit index (NFI = 0.91), incremental fit index (IFI = 0.90) and comparative fit index (CFI = 0.92). All values were adequate. On the other hand, the root mean square error of approximation (RMSEA) was 0.056, which is a value that demonstrates an adequate fit of the model and therefore its validity (Kock, 2014).

**Fig. 1:**
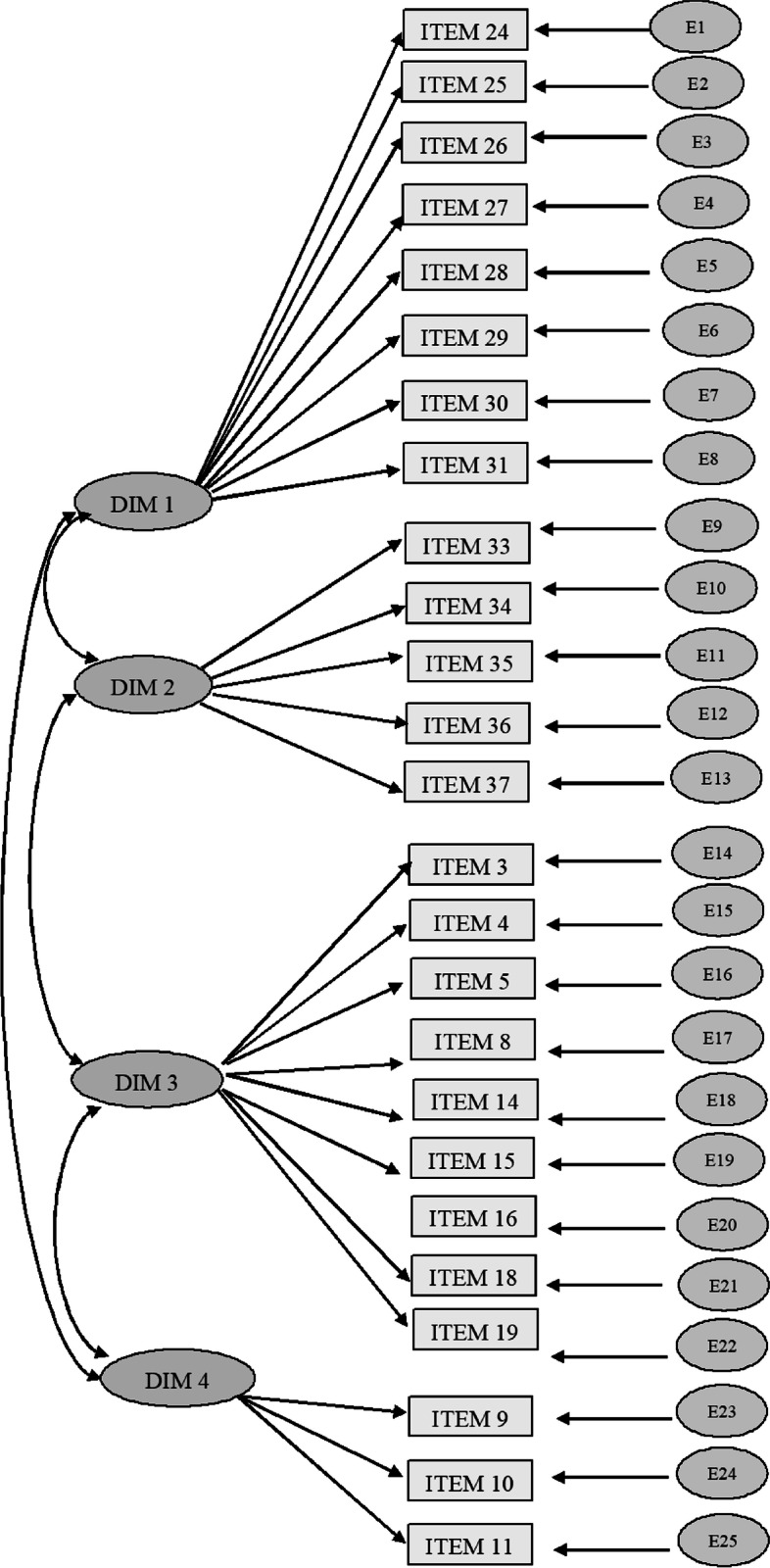
Graph of the theoretical model

Once the adjustments were made, the items whose standardised regression weights were less than 0.3 were eliminated, leaving the final questionnaire with 25 of the 39 initial items. The following graph shows the theoretical model (Fig. 1).

The standardised regression values for the associations established by the proposed structural model in relation to the emerged five factors and their indicators are presented in Table 1. All the associations examined were found to be positive and significant (*p* < 0.05).

**Table 1:** Standardised regression weights for scale items produced by the developed model

Item – factor association			Regression weights				SRW
			**Estimation**	**SE**	**CR**	**P**	**Estimation**
DIM1	< –-	MV	1.000				.993
DIM2	< –-	MV	1.000				.324
DIM3	< –-	MV	1.000				.224
DIM4	< –-	MV	1.000				.403
I24	< –-	DIM1	2.891	.197	14.681	***	.687
I25	< –-	DIM1	2.600	.178	14.643	***	.682
I26	< –-	DIM1	2.515	.174	14.437	***	.653
I27	< –-	DIM1	2.526	.185	13.682	***	.564
I28	< –-	DIM1	2.988	.215	13.925	***	.591
I29	< –-	DIM1	3.423	.251	13.619	***	.558
I30	< –-	DIM1	3.663	.265	13.804	***	.577
I31	< –-	DIM1	3.123	.242	12.882	***	.492
I37	< –-	DIM2	1.000				.539
I36	< –-	DIM2	2.218	.085	26.138	***	.940
I35	< –-	DIM2	2.258	.086	26.231	***	.947
I34	< –-	DIM2	2.020	.079	25.647	***	.903
I33	< –-	DIM2	2.257	.087	25.842	***	.917
I3	< –-	DIM3	1.000				.340
I5	< –-	DIM3	1.216	.106	11.457	***	.399
I4	< –-	DIM3	1.163	.095	12.232	***	.448
I8	< –-	DIM3	1.747	.120	14.565	***	.674
I14	< –-	DIM3	1.316	.092	14.348	***	.644
I15	< –-	DIM3	1.769	.125	14.095	***	.614
I16	< –-	DIM3	1.586	.115	13.846	***	.586
I18	< –-	DIM3	.877	.080	10.927	***	.369
I19	< –-	DIM3	1.756	.120	14.640	***	.685
I11	< –-	DIM4	1.000				.679
I10	< –-	DIM4	2.240	.134	16.685	***	.754
I9	< –-	DIM4	2.178	.129	16.914	***	.790

Note 1: SRW. Standardised regression weight; SE. Standard error; CR. Critical ratio

Note 2: *** Statistically significant differences at *p* < 0.005

Note 3: Dimension (Dim) 1 Sociability; Dimension 2 Transcendence; Dimension 3 Culture, and Dimension 4 Affection; MV (Multicultural Values)

The questionnaire consisted of sociodemographic questions and other specific questions on sociability. Specifically, there were 9 sociodemographic questions (sex, age, religion, whether the participant is a volunteer in any organisation, marital status, whether the participant has children, whether the participant has or has had dependents, whether the participant has pets, level of studies). The rest of the questionnaire contained 25 specific Likert scale questions with four response options (do not agree, slightly agree, agree, strongly agree) on multicultural values grouped in four blocks or dimensions: Dimension (Dim) 1: Sociability, which included the variables (items) I23, I24, I25, I26, I27, I28, I29, I30, I31; this variable focuses on the importance of empathy, harmonisation, how the participant is valued by others, the importance of truth, respect, doing good for others, multicultural coexistence and respect and care for sustainability; Dimension 2: Transcendence, which included the variables (items) I33, I34, I35, I36, I37, is measured by the opinion on the importance of believing in God and attending worship services, the identification of oneself as a believer, considering oneself a good believer, and taking actions for religious reasons; Dimension 3: Culture, which included the variables (items) I3, I4, I5, I14, I15, I16, I18, I18,I19, covers aspects such as reading habits, level of studies, ways of thinking, traditions and cultural events such as concerts, art, photography and others related to creativity; and Dimension 4: Affection, which included the variables I9, I10 and I11, and focuses on affection, physical contact, and the need to be surrounded by people.

Table 1 shows the indicator values that have the greatest influence with respect to each of the dimensions studied. Regarding the Sociability dimension, the variables with the highest regression weight and, therefore, with the greatest influence on the dimension were Items I24 [It is important to be empathetic to other people's problems] (*b* = 0.687; *p* < 0.005), I25 [Harmony is necessary] (*b* = 0.682; *p* < 0.005) and I26 [It is necessary to be considerate of others] (*b* = 0.653; *p* < 0.005). In the second dimension, the Transcendence dimension, the Items I33 [I believe in God] (*b* = 0.940; *p* < 0.005), I34 [I try to be a regular churchgoer] (*b* = 0.947; *p* < 0.005), I35 [Everyone knows that I am a believer] (*b* = 0.903; *p* < 0.005) and Item 36 [I am a good believer] (*b* = 0.917; *p* < 0.005) show high values that highlight this dimension as the dominant one with respect to the values. In reference to the Culture dimension, the most prominent items were I19, [I usually attend art events] (*b* = 0.685; *p* < 0.005) and I8 [I participate in the cultural traditions of my people] (*b* = 0.674; *p* < 0.005). Finally, in the Affection dimension, it is noteworthy that all the items have significant values: I9 [I consider myself affectionate towards others] (*b* = 0.790; *p* < 0.005), I10 [Others consider me affectionate] (*b* = 0.754; *p* < 0.005) and Item 11 [I need to have people around me] (*b* = 0.679; *p* < 0.005).

### Statistical analysis of the data

The statistical program IBM SPSS Statistics 25 was used to carry out the statistical analysis. Descriptive statistics were used to describe the data (frequencies, percentages, mean, and standard deviation). To meet the first objective and confirm the first hypothesis, a multivariate analysis of variance (MANOVA) was performed, in which the values related to prosocial behaviour were used as dependent variables and the variables of gender (masculine–feminine) and volunteer status were used as identification variables. The effect size was calculated with the partial eta square as a post hoc test using the Bonferroni test.

To meet the second objective and the second hypothesis, a multiple linear regression analysis was conducted (using the enter method). Each individual was entered as a dependent variable, with the predictor variables being the different dimensions linked to prosocial behaviour according to the values (transcendence, culture, affections, gender and participation in volunteering). To justify the method employed, the nonautocorrelation of the data was confirmed by the Durbin–Watson test, while the absence of multicollinearity was verified via the variance inflation factor.

## Results

### Sociability in relation to gender and volunteering

Table 2 shows multivariate analysis of variance (MANOVA) was used to examine the results related to the variables of gender and volunteering, as well as the interaction between them. The effect size was analyzed using eta-squared tests. According to the literature, eta-squared tests may indicate an effect size of *r* = 0.10, a mean effect size of *r* = 0.30, or a high effect size of *r* = 0.50 (Cohen, 1988; Field, 2009; Howell, 2007). Therefore, a value greater than 0.14 is considered to be high.

**Table 2:** Multivariate analysis of variance and effect size (η2) sums of aggregated scales for questionnaire on values for the promotion of prosocial behaviours by gender and volunteering

**Factors**		**M**	**SD**	**F**	***p***	***η***^**2**^
Sociability	Gender	3.5962	0.32464	23.979	0.000	0.015
	Volunteering activities	3.5962	0.32464	16.507	0.000	0.010
	Gender × Volunteering	3.5943	0.32555	14.749	0.000	0.028
Transcendence	Gender	2.4326	0.99616	2.022	0.155	0.001
	Volunteering	2.4326	0.99616	12.295	0.000	0.008
	Gender × Volunteering	2.4354	0.99575	5.027	0.002	0.010
Culture	Gender	2.8153	0.51532	18.327	0.000	0.012
	Volunteering	2.8153	0.51532	41.953	0.000	0.026
	Gender × Volunteering	2.8182	0.51695	19.024	0.000	0.035
Affection	Gender	3.1123	0.43231	9.292	0.002	0.006
	Volunteering	3.1123	0.43231	2.368	0.124	0.002
	Gender × Volunteering	3.1139	0.43180	2.436	0.063	0.005

The critical alpha level was adjusted for multiple testing to reduce the type III error (α). Thus, the α-value was divided by the number of pair comparisons for each ANOVA

This study aimed to assess the differences between participant groups based on their level of prosociality, considering their gender and whether they participated in volunteering. A two-way multivariate analysis of variance was used for each of the questionnaire items to determine their individual scores. To identify the effects of variance, a multivariate test was performed, which analyzed the relationship between the different levels of the same variable and between the levels of two different variables simultaneously (Andréu, 2011). The MANOVA tests showed significant differences and large effect sizes (> 0.14) for gender regarding sociability, significant differences for volunteering, and significant results (< 0.001) for the interaction between gender and volunteering. Female participants reported higher levels of sociability than male participants, and participants who volunteered had significantly higher scores on the sociability scale than non-volunteers. These findings support previous studies by Dunn et al. (2014) and Van Tongeren et al. (2018), which suggest that volunteering can have positive effects on social behavior. Furthermore, the interaction between gender and volunteering was found to be significant, indicating that the effect of volunteering on sociability differs by gender. Specifically, female participants who volunteered showed the highest scores on the sociability scale, while male participants who did not volunteer reported the lowest scores. These findings are in line with previous research by Smith et al. (2015), which found that gender and volunteering are important factors to consider when examining social behavior. In this study, a multivariate analysis of variance (MANOVA) was conducted to examine the relationship between gender and volunteering on prosocial behavior across cultures. The results showed significant associations between gender and volunteering on prosocial behavior (< 0.001). Specifically, large effect sizes (> 0.14) were found for the interaction between volunteering and gender, and for volunteering alone, although the effect size for gender was moderate. Women who participated in volunteering showed the highest social adaptation in cultural contexts. These results suggest that volunteering and gender are important predictors of prosocial behavior across cultures.

Furthermore, the multivariate analysis of variance revealed significant differences in prosocial behavior related to volunteering and its interaction with gender (< 0.001) in relation to transcendence. This finding is consistent with previous research by Gómez and Narváez (2019), who emphasized the importance of studying empathy, solidarity, emotional education, and prosocial moral criteria in juvenile offenders. Women who did not participate in volunteering showed the highest level of transcendental involvement. Finally, significant results were found regarding gender and being a volunteer (< 0.001) regarding Affection (the fourth dimension). Female volunteers showed greater affective development than male non-volunteers, as established by Gómez-Tabares (2019), who argued that prosocial tendencies and their relationship with empathy and self-efficacy beliefs for the regulation of affection are related to prosocial expressions.

Overall, these results suggest that gender and volunteering are important factors in predicting social behavior, and highlight the potential benefits of volunteering for promoting positive social outcomes. In summary, our results indicate that gender and volunteering have a significant impact on prosocial behavior across cultures. These findings have important implications for understanding the role of individual differences in promoting prosocial behavior in different cultural contexts.

### Predictive value of the different dimensions evaluated with respect to prosocial behaviour

The multivariate analysis technique of stepwise multiple linear regression was applied to establish how certain predictor or explanatory variables are related to the criterion variable. Specifically, the predictive value of prosocial behaviour measured through the sociability scale was calculated based on the variables of cultural development, experience of transcendence, feelings of affection and gender in relation to participation in volunteering.

The results from the multiple linear regression analysis comply with the approaches set out by the model, so it is considered valid (Aragón et al., 2015; Vilá et al., 2019), with the assumption of linearity verified (the partial scatter diagrams, see Figs. 4 and 5); the Durbin-Watson value between 1.5 and 2.5, specifically 1.959, which establishes a correct independence of errors; and adequate values of normality, homoscedasticity (*p* > 0.05), and noncollinearity (see Fig. 5 and Table 2). The multiple linear regression analysis suggested four models, with Model 4 offering the most significant explanatory power.

Before fitting the multiple linear regression model (Table 3), the linearity was verified, since the fact that two independent variables were correlated would have affected the model. None of the variables had a variance inflation factor (VIF) greater than 10, indicating that linearity was not a problem in the data.

**Table 3:** Stepwise multiple linear regression model for predicting sociability as a prosocial behaviour

Model^e^	*R*	*R*^*2*^	*R*^*2*^* adjusted*	Standard error estimated	d.f	*F*	*p* de *F*	Durbin-Watson
1	.432^a^	.187	.186	.29283	1	1564	.000	
2	.479^b^	.230	.229	.28508	2	1563	.000	
3	.488^c^	.238	.236	.28370	3	1562	.000	
4	.495^d^	.295	.293	.28250	4	1561	.000	1.959

^a^Predictor variables: (Constant), culture; ^b^Predictor variables: (Constant), culture, affection ^c^Predictor variables: (Constant), culture, affect, transcendence; ^d^Predictor variables: (Constant), culture, affect, transcendence, gender; ^e^ Dependent variable: sociability

The multivariate analysis technique of stepwise multiple linear regression was applied to establish how certain predictor or explanatory variables are related to the criterion variable that has been specified in the scale measuring sociability (for the analysis of prosocial behaviour). Specifically, the value was calculated using the variables of the scale measuring cultural development, the experience of transcendence, the feeling of affection, gender and participation in volunteering. The analyses allowed us to check the assumptions of the model except that of homoscedasticity, so it was tested with Leven's test to determine whether *p* > 0.05. The multiple linear regression analysis suggested 4 models, and the one with the greatest explanatory capacity was Model 4 (Table 1), with a good goodness of fit. Approximately 30% of the variance with respect to prosocial behaviour measured through the sociability scale is explained by the variables introduced in the model: cultural development, the experience of transcendence, the feeling of affection and gender in relation to participation in volunteering. This allows us to predict that the greater the involvement in transcendence, culture, affection and volunteering with respect to gender, the greater the development of prosocial behaviour.

**Fig. 2:**
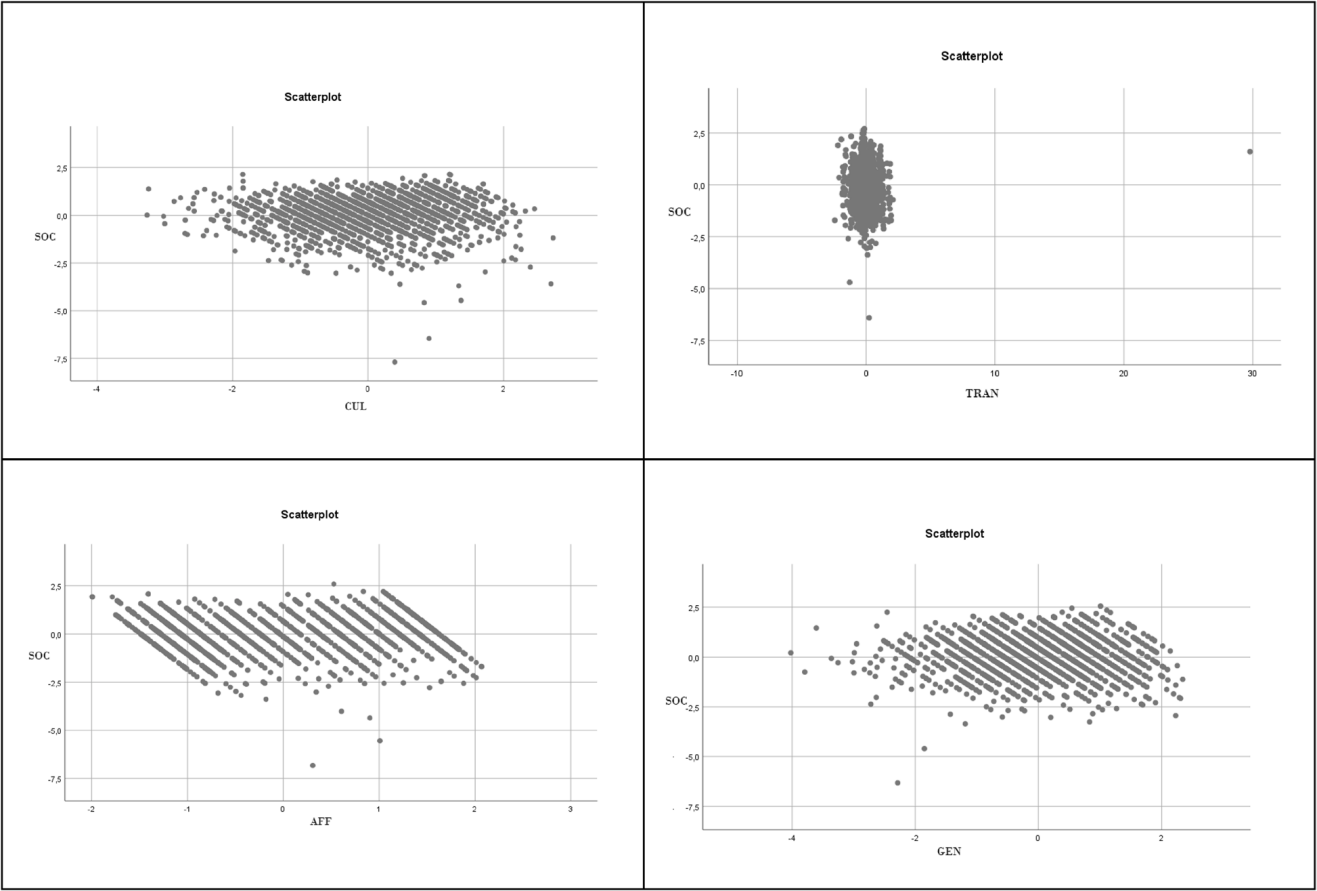
Partial regression plots of the sociability criterion variable (SOC), with each of the four predictor variables included in the model: The experience of transcendence, cultural development and the feeling of affection, gender with or without being a volunteer

**Fig. 3:**
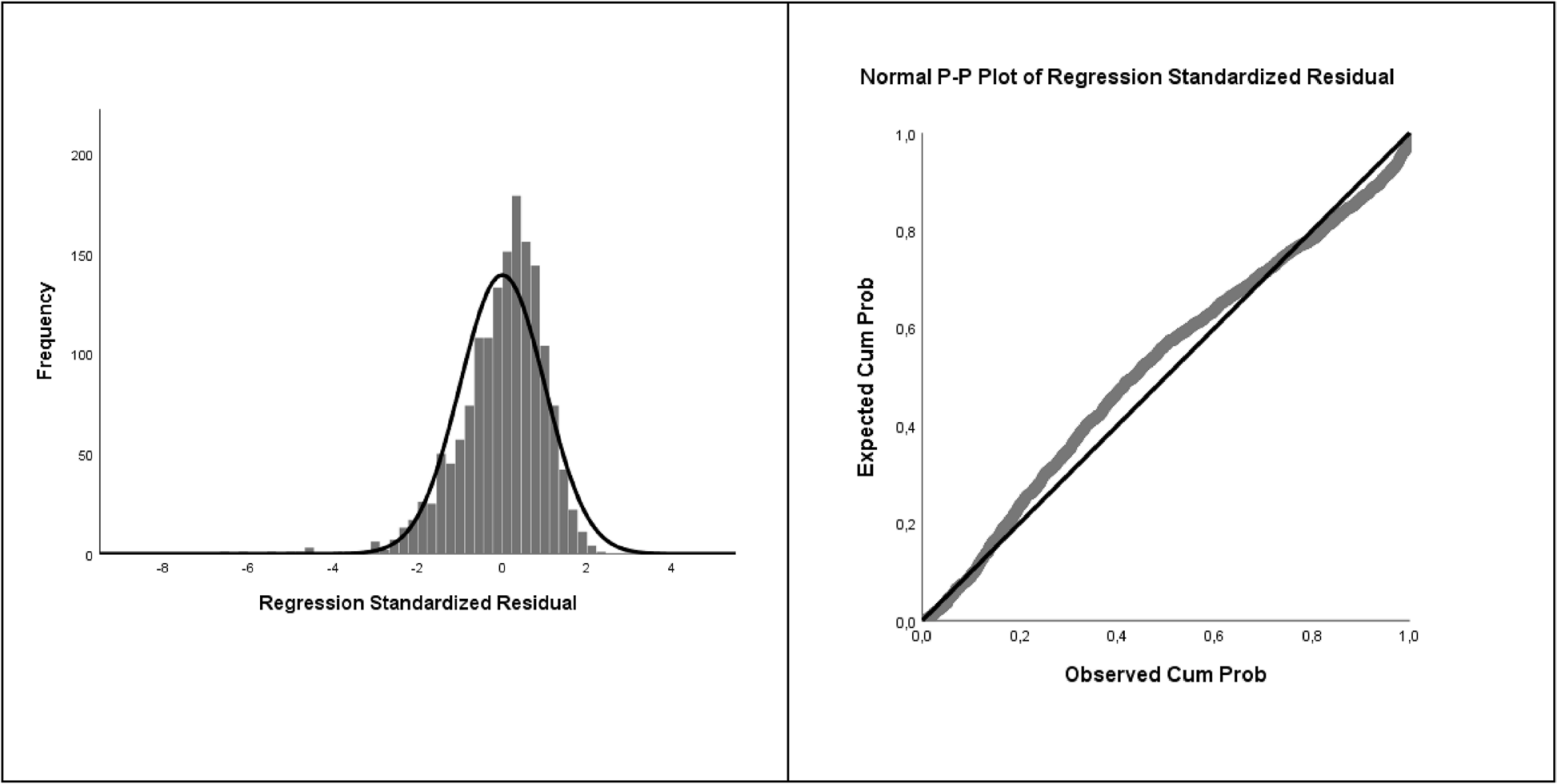
Normality assumption: Histogram and normal probability plot of the criterion variable

For a better understanding of the results, they are shown in the graphs below considering the four dimensions studied (Figs. 2 and 3).

Therefore, the results show that the sociability of an individual will be higher depending on their gender and according to their level of transcendence (religiosity or altruistic involvement). Transcendence is the strongest indicator of influence, followed by increased sociability. Greater cultural development and greater sociability increase slightly in the case of affective feelings or affective influencers, with volunteering according to gender being the least influential variable on prosocial behaviour, according to the results obtained in this regression.

Finally, the predictive equation derived by multiple linear regression analysis is shown. This equation makes it possible to predict sociability as a function of the variables studied based on the scores obtained. The predictive equation is as follows:

$$CDsoc = 2.829 + 0.911 CDtran+ 0.344 CDcult+ 0.201 CDaffec+ 0.083 Genvol$$

Based on Figs. 2 and 3 and Tables 2 and 3 and considering the variables that significantly influence each of the dimensions, the following conclusions can be drawn.

The results suggest that prosocial behaviour could be attributed to adequate sociability; therefore, respondents would consider the promotion of social values to improve it. This is also related to the prediction of the regression results, which confirmed that the promotion of social values (first dimension) encouraged positive prosocial behaviours.

## Discussion

How to achieve more social involvement of individuals to increase their prosocial behaviour, remains a complex and ongoing challenge. The study of social values helps us to determine which actions are conducive to this behaviour and thus allows us to encourage these actions aimed at the generation of socially positive attitudes. Likewise, it is essential to broaden the knowledge of the influences that other psychosocial characteristics of individuals and societies may have on such social behaviour. Shared transcendental ideas, common culture and the world of personal and interpersonal affections may be predictors that confirm their usefulness in the promotion of prosocial behaviour and the prevention of antisocial behaviour.

In relation to the hypotheses of the study, first, nonvolunteer women showed the highest values in terms of sociability, and having people under their care also had a positive influence on the results. Informal dedication to intrafamily care would make it difficult to develop formal volunteering activities, but in no way would it mean an absence of prosocial involvement. In this sense, the assessment of prosociality made only by considering the environment external to the different family nuclei (NGOs, associations, etc.) could be questioned because, in the household environment, a series of social functions are conducted that go unnoticed, despite being essential for society, such as caring for sick family members or bringing up minors. These activities are mainly carried out by women in many societies. According to the results of the study by Craig (2006), in many cultures, in comparison with fatherhood, motherhood implies more general responsibility in the management of care, and this gender difference in the amount and nature of care applies even when women work full-time. This was observed when analysing the results of the eta square test (Coe & Merino, 2003; Lakens, 2013; Richardson, 2011) in relation to culture, which indicates that the recognition and promotion of cultural values can enhance prosocial behaviour. In this study, nonvolunteer women with dependents under their care show the highest values in relation to prosocial behaviour by promoting cultural activities, which coincides in the study with the relevance of the sociability dimension. The positive assessment by women of common social expressions may have an important implicit social function in the cultural dimension rather than being a mere expression of its cultural factor. Hypothesis 1 is therefore confirmed; that is, the results obtained indicate that prosocial behaviour varies according to gender and whether one participates in volunteering.

In the predictive analysis developed to study prosocial behaviour, to meet the second objective and the second hypothesis of the study, we found that the values of the Transcendence dimension are related to volunteering, showing a high predictive value with an especially high variability (> 40%), and to religion, one of the factors influencing the relevance of this dimension. In this dimension, no gender differences were detected, and as in the previous dimension, participants’ not being a volunteer and having people under their care had a considerable influence on the results. This confirms the study by Balabanian and Lemos (2020) and their idea that mental models based on beliefs about the world have a decisive influence on prosocial tendencies.

It is worth noting that both social volunteering and religion, not spirituality, are objectively formal social activities. Likewise, it is interesting to highlight, in the search for values that promote prosociality, the significance of relational community values (social, cultural, and transcendental) over affective values (more linked to the world of interpersonal relationships and feelings). As they can be the interaction between empathy and moral pride (Ortiz Baron et al., 2018), social recognition and reputation increase fairness and trust (Duradoni et al., 2018), and the development of a positive reputation can be an asset that will lead to better results. Perhaps, to achieve greater prosociality in individuals, formal actions of active and continuous participation should be encouraged, not only membership or affiliation to a social action group. As Futamura (2018) argues, extraordinary prosocial actions are highly valued when they are accompanied by ordinary prosocial behaviours, so they need to be performed regularly and not just on an exceptional basis. In addition, to achieve greater social involvement, it might be more advantageous to promote a more transcendent approach to social reality rather than a more ordinary or common approach, pursuing more universal values in terms of motivating prosocial actions.

Regarding the last dimension studied, which refers to Affection, women who look after their dependent relatives show higher positive values. This is linked to the prediction that more prosocial behaviour is associated with those who have dependents under their care, although the interaction does not have significant differences because this care is not visible. Some studies support that nudges are not effective when the target behaviour is not perceived as common or is not visible, such as the relation of reinforcement of parental values to young children’s prosocial behaviours (Tyers, 2018). This is consistent with the claim by Baez et al. (2017) that women are more empathetic when they are aware that they are being evaluated and that gender does not seem to be a good predictor of empathy.

Among the main limitations of this study was the difficulty in accessing the sample, which, being nonprobabilistic, has an impact on the limits of the analyses used, although the time required for data collection was considered reasonable. It can be concluded from this that the results of the study allow a multidimensional analysis to be carried out. It is therefore considered necessary to extend the sample to other regions of Spain to obtain significant results from the data collected using the questionnaire. This would also allow comparisons to be made with other regions. Another limitation is that opinion studies with participants are not completely free of bias, although they were controlled by not informing participants of the purpose of the study to prevent invalid responses and to reduce the influence of social desirability.

Gender differences and volunteer status (being or not being a volunteer) as well as the analysed constructs were revealed. However, it is imperative to perform an in-depth analysis of the variables that stood out in this study, such as personal (i.e., age), family (i.e., children, or dependent elderly family members), religion, and social variables that contribute to the improvement of prosocial behaviour based on the enhancement of values. Some of these variables were exposed for their importance, but a more exhaustive analysis could lead to more useful and complete results.

### Practical implications

In view of the results, a number of possible practical actions aimed at promoting prosociality can be proposed:

Increasing the importance of education in social sciences and psychology within the school curriculum, seeking an education that focuses on social and emotional competences in which prosocial behaviour is encouraged. The transcendental aspects of life should be emphasised with powerful concepts such as love, solidarity, the common good, community, etc.

Taking into account gender differences when promoting volunteering, enhancing the value of women in this area through the implementation of egalitarian actions with regard to children’s upbringing and the care of dependent elderly family members.

Having confirmed the significance of engaging in volunteering activities, more early awareness-raising efforts could be made in schools to encourage future participation in volunteering. For instance, local guides could be created and serve as a catalogue of the different volunteering organisations in the area and the tasks or activities involved.

Moreover, to conduct further research on this topic, it is necessary to acquire new and increasingly specific measurement tools. The creation of new measurement instruments such as the one by Ruiz-Ordóñez et al. (2022) to measure the impact of a service-learning methodology on both civic values and civic attitudes is a good example of specialisation in the search for understanding and promoting prosocial behaviour.

## Conclusion

The results obtained in the study allow the following conclusions to be drawn: having grouped the values that could promote prosocial behaviour, prosociality was found in the values linked to concrete actions, both formal (participation in actions of volunteering groups or religious organisations) and informal (intervention in the care of family members). The role of women as socialising agents stands out owing to a greater relevance of values for women that facilitate coexistence under common norms. Prosocial behaviour, and therefore the sociability of the individual, can be improved through transcendence, highlighting their interrelation of service to others, their altruism and the ability to improve feelings of affection in connection with the promotion of cultural development actions. To promote greater prosociality, cultural actions that involve meeting with others and social relationships as well as involvement in volunteer activities should be promoted, mainly in men. To do this, we must implement governmental, educational, cultural, ideological and social actions through awareness-raising and training programmes and projects that encourage the implications of caring for others for the promotion of prosocial behaviour, directly influencing future generations. This is necessary if we want to improve prosociality and the development of the individual both at an intrinsic and extrinsic level.

It is noteworthy that prosociality does not necessarily correlate with volunteering, at least in the case of nonvolunteer women with dependents. There are prosocial behaviours in formal (institutionalised) settings such as the home that may go unnoticed, so it should be borne in mind that the family setting is itself a social setting.

The Transcendent dimension fosters prosociality. Anything that forces individuals to physically and mentally step beyond themselves (out of their own daily routines, out of the habits that give them security) in search of "the other"—the need for others, encounters with different people and cultures—will encourage prosociality. Therefore, the ability to look outside oneself and to seek the good of others, should be a desirable goal in cultural education if we want to achieve viable and sustainable communities.

To achieve greater social involvement, it might be more advantageous to promote a more transcendent vision of social reality rather than a more materialistic one, motivating individuals to look for more universal values. For example, an action motivated by the good that is done to the elderly when they are visited would be more effective than justifying it in terms of how good the visitor would feel.

## Data Availability

Data provided by the informants in the study is only available to the authors.

## Appendix

### Dim. 1: Sociability

24. It is important to be empathetic to other people's problems.

25. Harmony is necessary.

26. It is necessary to be considerate of others.

27. Truth is important in my life.

28. Respect others if they are different.

29. It is more important to do good than to be right.

30. It is necessary to encourage coexistence between different cultures.

31. I am concerned about depleting resources or causing damage to the environment.

### Dim. 2: Transcendence

33. I believe in God.

34. I try to be a regular churchgoer.

35. Everyone knows that I am a believer.

36. I am a good believer.

37. I am supportive for religious reasons.

### Dim. 3: Culture

3. I enjoy reading.

4. I enjoy learning new things.

5. I tend to think carefully and thoroughly about things.

8. I participate in the cultural traditions of my people.

14. Culture makes me a better person.

15. I like concerts.

16. I like cinema and theatre.

18. I like graphic arts.

19. I usually attend art events.

### Dim. 4: Affection

9. I consider myself caring towards others.

10. Others consider me caring.

11. I need to have people around me.

## Abbreviations

EU: European Union
GDPR: General Data Protection Regulation
LOPDGDD: Ley Orgánica de Protección de Datos y Garantía de Derechos Digitales (Organic Law on Data Protection and Guarantee of Digital Rights)
IBM SPSS: International Business Machines; Statistical Package for Social Sciences
MANOVA: Multivariate analysis of variance
ANOVA: Analysis of variance
SOC: Sociability
TRA: Transcendence
CUL: Culture
AF: Affection
SEM: Structural equation modelling
NFI: Normalised fit index
IFI: Incremental fit index
CFI: Comparative fit index
RMSEA: Root mean square error of approximation
Dim: Dimension
VIF: Variance inflation factor
η2: Effects of size
NGOs: Nongovernmental organisations
r: Effect of eta square tests
SRW: Standardised regression weight
SE: Standard error
CR: Critical ratio
***: Statistically significant differences at the level of *p* < 0.005.
MV: Multicultural Values
M: Mean
SD: Standard deviation
R: Correlation coefficient
R^2^: Coefficient of determination
d.f: Degrees of freedom
F: Statistic quotient of two variances
*p* de F: Predictor significant correlation to your model
*p*: Statistically significant differences at the level of *p* < 0.001

## Code availability

The data presented in this study are available on request from the Principal Researcher: MCO-G, mcolmos@ugr.es.
